# Supervised sequential pattern mining of event sequences in sport to identify important patterns of play: An application to rugby union

**DOI:** 10.1371/journal.pone.0256329

**Published:** 2021-09-23

**Authors:** Rory Bunker, Keisuke Fujii, Hiroyuki Hanada, Ichiro Takeuchi

**Affiliations:** 1 Graduate School of Informatics, Nagoya University, Nagoya, Aichi, Japan; 2 RIKEN Center for Advanced Intelligence Project, Tokyo, Japan; 3 Department of Computer Science, Nagoya Institute of Technology, Nagoya, Aichi, Japan; Western Norway University of Applied Sciences, NORWAY

## Abstract

Given a set of sequences comprised of time-ordered events, sequential pattern mining is useful to identify frequent subsequences from different sequences or within the same sequence. However, in sport, these techniques cannot determine the importance of particular patterns of play to good or bad outcomes, which is often of greater interest to coaches and performance analysts. In this study, we apply a recently proposed supervised sequential pattern mining algorithm called safe pattern pruning (SPP) to 490 labelled event sequences representing passages of play from one rugby team’s matches in the 2018 Japan Top League season. We obtain patterns that are the most discriminative between scoring and non-scoring outcomes from both the team’s and opposition teams’ perspectives using SPP, and compare these with the most frequent patterns obtained with well-known unsupervised sequential pattern mining algorithms when applied to subsets of the original dataset, split on the label. From our obtained results, line breaks, successful line-outs, regained kicks in play, repeated phase-breakdown play, and failed exit plays by the opposition team were found to be the patterns that discriminated most between the team scoring and not scoring. Opposition team line breaks, errors made by the team, opposition team line-outs, and repeated phase-breakdown play by the opposition team were found to be the patterns that discriminated most between the opposition team scoring and not scoring. It was also found that, probably because of the supervised nature and pruning/safe-screening mechanisms of SPP, compared to the patterns obtained by the unsupervised methods, those obtained by SPP were more sophisticated in terms of containing a greater variety of events, and when interpreted, the SPP-obtained patterns would also be more useful for coaches and performance analysts.

## Introduction

Large amounts of data are now being captured in sport as a result of the increased use of GPS tracking, optical, and video analysis systems, as well as enhancements in computing power and storage. There is great interest in making use of this data for performance analysis purposes. A wide variety of methods have been used to analyze sports data, ranging from statistical methods to, more recently, machine learning, deep learning and data mining techniques.

Among the various analytical frameworks available in sports analytics, in this paper, we adopt an approach to extract events from sports matches and analyze sequences of events. The most basic events-based approach is based on the analysis of the frequencies of events, which can be used as performance indicators [1]. Alternatively, by comparing the frequency of each event in sequences with positive outcomes (winning, scoring points, etc.) with the frequency of each event in sequences with negative outcomes (losing, conceding points, etc.), one can investigate which events are commonly associated with these outcomes. However, frequency-based analyses have drawbacks in that the information contained in the order of events cannot be exploited.

In this study, we consider sequences of events, and refer to a partial sequence of events as a sequential pattern, pattern of play, subsequence, or simply a pattern. In sports, the occurrence of certain events in a particular order often has a strong influence on outcomes, so it is useful to use patterns as a basic analytical unit. Invasion sports, e.g., rugby, soccer and basketball, have many events and patterns that occur very frequently and repeatedly. However, although there may be a paucity of events that are important for scoring, these are the patterns that are of greater interest to coaches and performance analysts. For instance, in soccer, a pattern consisting of an accurate cross followed by a header that is on target will occur much less frequently than a pattern consisting of repeated passes between players, but the former pattern is likely to be of much greater interest to coaches and performance analysts because there is a good chance that the pattern may lead to a goal being scored.

The computational framework for finding patterns from sequential data that have specific characteristics is known as sequential pattern mining (SPM) in the field of data mining. The most basic problem setup in SPM is to enumerate frequent patterns, which is called frequent SPM. Although the total number of patterns (i.e., the number of ordered sequences of all possible events) is generally very large, it is possible to efficiently enumerate patterns that appear more than a certain number of times by making effective use of branch-and-bound techniques. In the field of machine learning, frequent SPM is categorised as an unsupervised learning technique.

When applying frequent SPM to data from sport, there are several options. The first option is to simply extract the frequent patterns from the entire dataset. The drawback of this approach is that it is not possible to determine whether a particular pattern leads to good or bad outcomes. The second option is to split the dataset into a “good-outcome” dataset and a “bad-outcome” dataset, and apply frequent SPM to each dataset. The third option is to apply frequent SPM to the entire dataset in order to identify frequent patterns, and then create a machine learning model that uses these patterns as features to predict whether the pattern is associated with good or bad outcomes. A disadvantage of the second and third options is that the pattern extraction process is conducted separately from the process that associates patterns with outcomes.

Unlike unsupervised SPM, supervised SPM directly extracts patterns that are associated with good or bad outcomes. Roughly speaking, by using supervised SPM, we can identify patterns that differ in frequency according to the outcomes in a more direct manner.

### Related work

#### Sequential pattern mining (SPM)

Sequential pattern mining (SPM) [2] involves discovering frequent subsequences as patterns from a database that consists of ordered event sequences, with or without strict notions of time [3]. Originally used to analyze biological sequences [4–7], SPM methods have also been applied for other purposes including XML document classification [8], keyword/key-phrase extraction [9–11], as well as next item/activity prediction and recommendation systems [12–17]. For an overview of the SPM field, we refer the reader to [18].

A recent area of interest in SPM has been high-utility SPM, which built on the idea of high-utility pattern (itemset) mining [19] by taking into consideration the utility of patterns. For instance, [20] provides an example where in market basket analysis, although a diamond may sell much less frequently than an egg, it is of much higher utility (profit) and is thus of greater interest to a business. An early study was that of [21], who defined the high-utility SPM problem and proposed a method for it called USpan. Other proposed high-utility SPM methods include HUS-SPAN [22], which was expanded on by [23], who used pruning methods to decrease the required pattern search space in order to improve efficiency in terms of run-time and the number of candidate patterns generated. Scalable high-utlity SPM methods that can be applied to big data (e.g., IoT) have been proposed by [24], who introduced a scalable Spark-based platform, and [25], who proposed a Hadoop-based high fuzzy utility pattern mining (HFUPM) algorithm.

In market basket analysis applications of high-utility SPM, per-item prices or profits can be used as utility values in order to then determine which patterns are interesting; however, in sport, it is difficult to determine explicit *a priori* values for events or patterns. Thus, to overcome this problem, in this study, by assigning outcome labels to sequences based on scoring outcomes and taking a discriminative approach that employs supervised learning, we can instead identify interesting patterns by determining their *a posteriori* values.

One of the first (standard) SPM algorithms to be proposed was GSP [26], which was based on the A-priori algorithm proposed by the same authors [27]. Algorithms including SPADE [28], SPAM [29], and PrefixSpan [30] were later proposed to address some of the limitations that were identified with GSP. PrefixSpan is categorized as a pattern-growth algorithm, since it grows a tree structure of patterns extending from a pattern with a single event at its base, and adds greater numbers of events to the patterns in each of its descendent nodes for all possible patterns in a database. More recently, CM-SPAM and CM-SPADE [31] as well as Fast [32] have been proposed to provide further improvements in computational efficiency and speed compared to the original SPAM and SPADE algorithms. It should be noted that all of the above-mentioned SPM methods are unsupervised methods, which are applied to unlabelled sequences.

**Fig 1:**
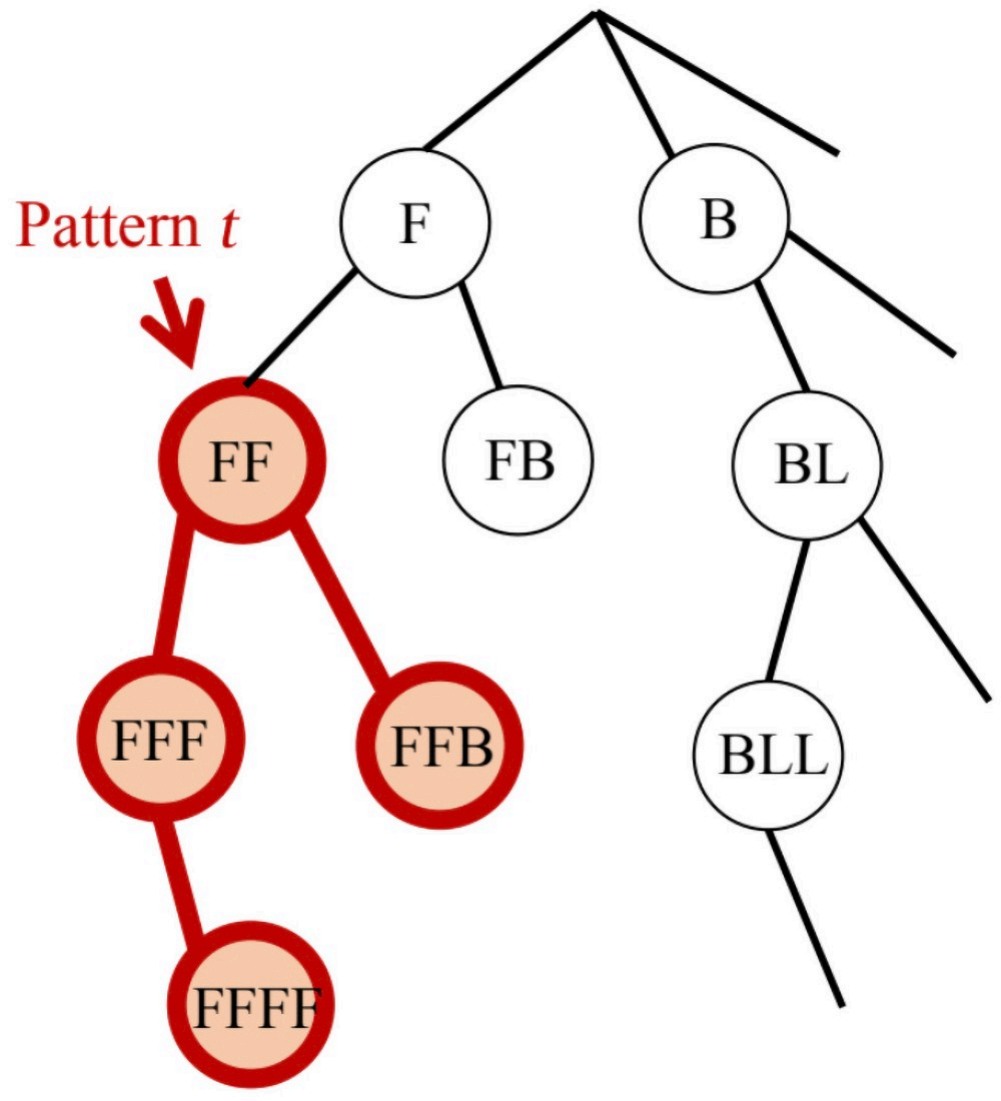
SPP pruning. One of the mechanisms of SPP identifies and removes patterns that do not contribute to the model before performing the optimization. For example, if pattern *t* does not satisfy the pruning criterion specified in [34], the sub-tree below pattern *t* is deleted.

Safe pattern pruning (SPP), proposed by [33, 34], combines a convex optimisation technique called safe screening [35] with SPM. SPP is supervised, and can be applied to labelled sequences. SPP uses PrefixSpan to grow the initial pattern tree, and redundant patterns are then removed using a specific pruning criterion (see [33, 34] for more details on this pruning criterion). In particular, the tree structure grown by PrefixSpan is pruned in such a way that if a node corresponding to a particular pattern is pruned, it is guaranteed that all patterns corresponding to its descendant nodes are not required for the predictive SPP model (Fig 1).

In the SPP method, each pattern is multiplied by a weight, and these weights are calculated by solving an optimization problem, as will be described later in this paper. The magnitude of the weight of a pattern reflects the degree to which that pattern discriminates between positive and negative outcomes (labels). As mentioned, the SPP method also incorporates safe screening, which eliminates redundant weights that are guaranteed to be non-discriminative in the optimal solution (i.e., will have a weight value of zero). SPP has previously been applied to datasets consisting of animal trajectories [34]; however, compared with animal trajectories, data in sport often contains a greater diversity of events.

#### Application of SPM methods in sport

Some previous studies have applied unsupervised SPM methods to sport, and these are summarized in Table 1. These prior studies have focused mainly on the identification, interpretation and visualization of sequential patterns. CM-SPAM was applied for performing technical tactical analysis in judo by [36], while sequential data that was obtained using trackers was used to test for significant trends and interesting sequential patterns in a single cyclist’s training regime over an extended period of time by [37]. In the context of team sport (soccer), Decroos et al. [38] combined clustering and CM-SPADE using a five-step approach that is summarized in the fifth column of Table 1, and the authors provided a ranking function that allowed the user (e.g., a coach) to assign higher weights to events that are more relevant. For instance, the authors note that, despite their frequency, normal passes are not as of much relevance to coaches as shots and crosses.

**Table 1:** Prior studies that have applied sequential pattern mining techniques in sport.

Study	Sport	Model Used	Model Type	Summary of Approach	Evaluation Metrics
[36]	Judo	CM-SPAM	Unsupervised	Identified patterns with SPM for the tactical analysis of judo techniques	Support
[37]	Cycling	SPADE	Unsupervised	Applied SPADE to identify frequent sequential patterns, calculated interestingness measures (p-values) for these frequent patterns, and visualized these patterns for increasing/decreasing daily and duration trends	Support, permutation test p-values
[38]	Soccer	CM-SPADE	Unsupervised	Clustered phases based on spatio-temporal components, ranked these clusters, mined the clusters to identify frequent sequential patterns, used a ranking function (weighted support function)—in which a coach can assign higher weights to more relevant events—to score obtained patterns, and then interpreted the obtained patterns	Support (events were weighted according to their relevance based on the judgement of the user), and identified the top-ranked frequent sequences in the clusters

#### Analysis of sequences in rugby union

In the sport of rugby union (hereafter referred to simply as rugby), some prior studies have considered sequences of play by analyzing their duration. For example, [39] studied the durations of sequences of plays leading to tries at the 1995 Rugby World Cup (RWC), and [40] found that, at the 2003 RWC, teams that were able to create movements that lasted longer than 80 seconds were more successful. More recently, [41] applied K-modes cluster analysis to sequences of play in rugby, and found that scrums, line-outs and kick receipts were common scenarios that led to tries being scored in the 2018 Super Rugby season. By employing convolutional and recurrent neural networks, [42] aimed to predict the outcomes of sequences of play (e.g., whether the sequence of play ended with territory gain, retaining of possession, scoring of a try, or conceding/being awarded a penalty), based on the order of events and their on-field locations.

### Motivation and contributions

In this study, we apply SPP, a supervised SPM method, to data consisting of event sequences from all of the matches played by a professional rugby union team in their 2018 Japan Top League season. The present study is motivated by the fact that, although SPM methods have been applied to sport, only unsupervised SPM methods appear to have been used to date. In addition, no form of SPM method, unsupervised or supervised, appears to have been applied to analyze sequences of play in rugby.

We compare the most discriminative SPP-obtained patterns (subsequences) with the most frequent patterns obtained by well-known unsupervised SPM methods (PrefixSpan, GSP, Fast, CM-SPADE and CM-SPAM), where the unsupervised SPM methods are assumed to possess knowledge of which sequences contain scoring events (i.e., when the unsupervised methods are applied to label-based partitions of the original labelled data).

The main contributions of this study are in the comparison of the usefulness of supervised and unsupervised SPM methods when applied to event sequence data in sport, the application of a supervised SPM method to event sequence data in sport, and the application of a SPM method to analyze sequences of play in rugby.

### Notation

Sequences consist of ordered events drawn from a set of *m* unique event symbols, denoted $S\;≔\;\{s_{1},\ldots ,s_{m}\}$. Let *n* denote the number of sequences in the dataset. The sets of sequences with labels 1 and -1 are denoted by $G_{+},G_{-}\subseteq \left[n\right]$, and are of size $n_{+}\;≔\;|G_{+}|,n_{-}≔\left|G_{-}\right|$, respectively. SPP takes as input a set of *n* labeled sequences:

$$\begin{array}{c} {\left\{\left(g_{i},y_{i}\right)\right\}}_{i\in [n]}, \end{array}$$

where ***g***_*i*_ represents the *i*th sequence/passage of play. Each sequence ***g***_*i*_ takes a label from *y*_*i*_ ∈ {±1} and can be written as

$$\begin{array}{c} g_{i}=\left\langle g_{i1},g_{i2},\ldots ,g_{iT(i)}\right\rangle ,i\in \left[n\right], \end{array}$$

where *g*_*it*_ is the *t*th symbol of the *i*th sequence, which takes one of the event symbols in S, and *T*(*i*) is the length of the *i*th sequence (i.e., *T*(*i*) is the number of events in sequence ***g***_*i*_). Patterns of play are denoted by ***q***_1_, ***q***_2_, …, each of which is also a sequence of event symbols:

$$\begin{array}{c} q_{j}=\left\langle q_{j1},q_{j2},\ldots ,q_{jL(j)}\right\rangle ,j=1,2,\ldots , \end{array}$$

where *L*(*j*) is the length of pattern ***q***_*j*_ for *j* = 1, 2, …. The presence of subsequence ***q***_*j*_ in sequence ***g***_*i*_ is denoted by ***q***_*j*_ ⊑ ***g***_*i*_. The set of all possible patterns contained in any sequence {***g***_*i*_}_*i*∈[*n*]_ is denoted as $Q={\left\{q_{i}\right\}}_{i\in [d]}$, where *d* is the number of possible patterns (Q is very large in general, which is why the pruning and safe-screening mechanisms of SPP are useful).

## Materials and methods

### Data

**Fig 2:**
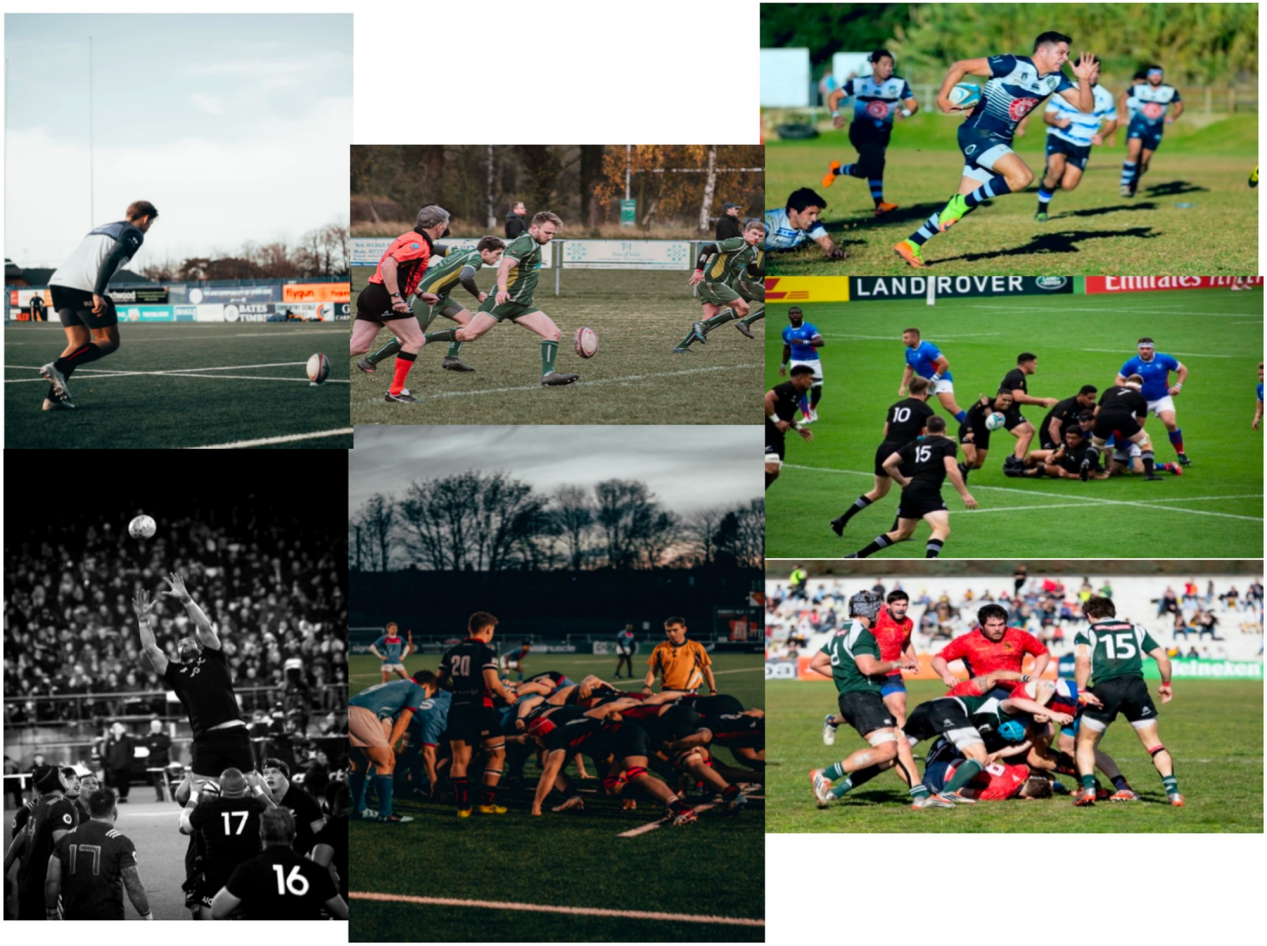
Key events in rugby matches. The photographs used as the original images are listed in parentheses. All of them are licensed under the unsplash.com license (https://unsplash.com/license). Top left: Kick at goal (https://unsplash.com/photos/xJSPP3H8XTQ); Bottom left: Line-out (https://unsplash.com/photos/CTEvFbFpVC8); Center top: Kick restart/Kick-off (https://unsplash.com/photos/OMdge7F2FyA); Center bottom: Scrum (https://unsplash.com/photos/y5H3_7OobJw); Top Right: Line break (https://unsplash.com/photos/XAlKHW9ierw); Middle Right: Beginning of a phase (https://unsplash.com/photos/fqrzserMsX4); Bottom Right: Breakdown (https://unsplash.com/photos/WByu11skzSc).

We obtained XML data generated from video that was tagged in Hudl Sportscode (https://www.hudl.com/products/sportscode) by the performance analyst of one of the teams in the Japan Top League competition (the team is not named for reasons of confidentiality). Written consent was obtained to use the data for research purposes. Seasons are comprised of a number of matches, matches are made up of sequences of play, which are, in turn, comprised of events. Our dataset consisted of all of this particular team’s matches in their 2018 season for each of the opposition teams they faced. These matches consist of passages of play (i.e., sequences of events), however, rules need to be specified to decide the point at which these passages of play start and end. Initially, each match in the original dataset was one long sequence of events. One approach that we considered initially, which we used on other datasets, was to label match sequences based on whether the team won or lost the match. However, in our initial experiments, this did not produce interesting results since it is obvious that a greater number of scoring events will occur within match sequences labeled with wins, and so the discriminative patterns identified largely contained only contain these scoring events. Therefore, we generated a more granular dataset by specifying rules to delimit the match sequences into sequences representing individual passages of play (these rules are described in more detail in the following subsection). Each sequence was comprised of a series of events from 24 unique events (12 unique events for the team and opposition teams), based on the events the analyst had tagged in SportsCode. These events are listed in Table 2, and some are also depicted in Fig 2 (the XML data also contained a more granular level of data with a greater number of unique events, however, in order to reduce computational complexity, the higher level of granularity was considered).

**Table 2:** Unique events in the original XML data. Events prefixed by “O-” are performed by the opposition team; those that are not are performed by the team.

event ID	event	event description
1	Restart Received	Team receives a kick restart made by the opposition team
2	Phase	Period between breakdowns (team in possession of the ball)
3	Breakdown	Team player is tackled, resulting in a ruck
4	Kick in Play	Kick within the field of play (rather than to touch) made by the team
5	Penalty Conceded	Team gives away a penalty, opposition may re-gain possession
6	Kick at Goal	Team attempts kick at goal
7	Quick Tap	Quick restart of play by the team following a free kick awarded to them
8	Line-out	Ball is thrown in by the team
9	Error	Mistake made by the team, e.g., lost possession, forward pass, etc.
10	Scrum	Set piece in which the forwards attempt to push the opposing team off the ball
11	Try Scored	Team places the ball down over opposition team’s line (five points)
12	Line Breaks	Team breaches the opposition team’s defensive line
13	O-Restart Received	Opposition team receives a kick restart made by the team
14	O-Phase	Period between breakdowns (opposition team in possession of the ball)
15	O-Breakdown	Opposition player is tackled, resulting in a ruck
16	O-Kick in Play	Kick within the field of play (rather than to touch) made by the opposition team
17	O-Penalty Conceded	Opposition team gives away a penalty, team may re-gain possession
18	O-Kick at Goal	Opposition team attempts kick at goal
19	O-Quick Tap	Quick restart of play by the opposition team following a free kick awarded to them
20	O-Line-out	Ball is thrown in by the opposition team
21	O-Error	Mistake made by the opposition team, e.g., lost possession, forward pass, etc.
22	O-Scrum	Set piece in which the forwards attempt to push the team off the ball
23	O-Try Scored	Opposition team places the ball down over the team’s line (five points)
24	O-Line Breaks	Opposition team breaches the team’s defensive line

### Methods

#### Delimiting matches into sequences

**Fig 3:**
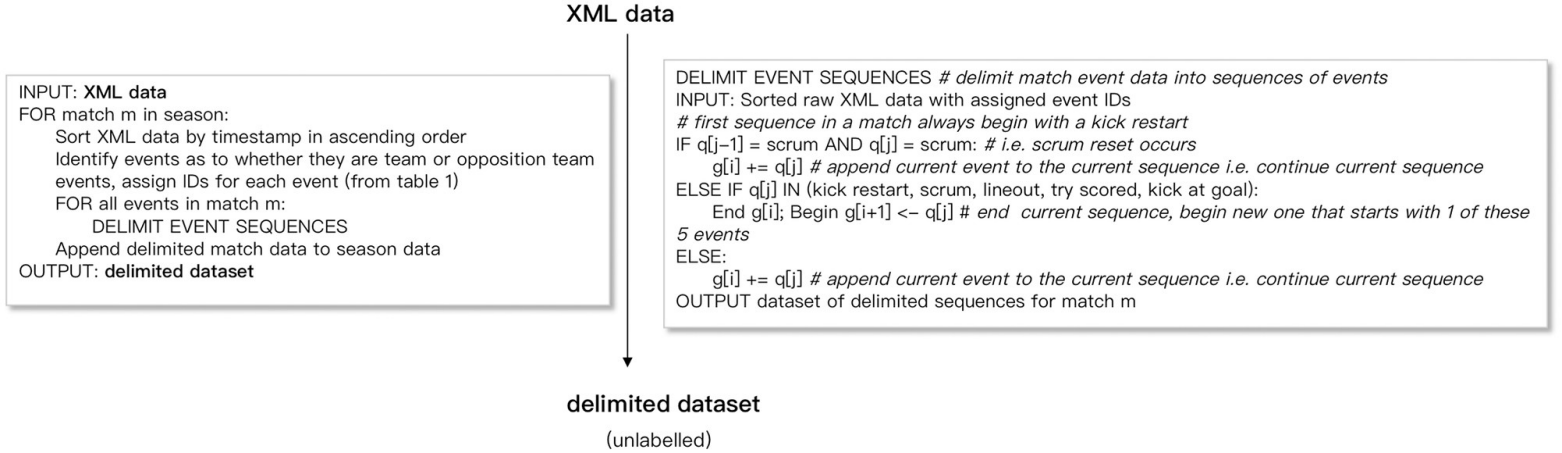
Converting XML files into labeled sequences. Illustration of the procedures and specified rules to delimit the raw XML data files into passage of play event sequences labeled with scoring or conceding outcomes.

First, each match sequence was delimited into passage of play event sequences (Fig 3). The rules to delimit matches into passages of play should ideally result in passages of play that begin and end at logical points in the match, e.g., when certain events occur, when play stops, or when possession changes (e.g., [43]), and should result in sequences which are neither overly long nor overly short. In this study, a passage of play was defined to start with either a kick restart, scrum, or line-out. These three events result in play temporarily stopping and therefore represent natural delimiters for our dataset. When a kick restart, scrum (except for a scrum reset where a scrum follows another scrum), or line-out occurs, this event becomes the first event in a new event sequence; otherwise, if a try is scored or a kick at goal occurs, a new passage of play also begins. Applying these rules (also shown in Fig 3) resulted in a delimited dataset consisting of 490 sequences, each made up of events listed in Table 2. At this stage, the delimited dataset was unlabelled, and the scoring events (try scored, kick at goal) for the team and opposition teams were contained in the sequences.

#### Experimental dataset creation and comparative approach

The delimited sequence data described above was then divided into two datasets. In the first, which we call the scoring dataset, we consider the case in which the sequences are from the team’s scoring perspective. In this dataset, the label *y*_*i*_ = +1 represents points being scored or attempted by the team. Note that while a try scored was certain in terms of points being scored, a kick at goal (depicted in the top-left of Fig 2) is not always successful and therefore may not result in points being scored. In our data, only the kick at goal being attempted (event id 6) was available—not whether the goal was actually successful or not. However, since it is more important to be able to identify points-scoring opportunities than whether or not the kick was ultimately successful (which is determined by the accuracy of the goal kicker), we assumed that all kicks at goal resulted in points being scored. In the scoring dataset, the label *y*_*i*_ = +1 was assigned to sequence *i* if a try was scored or a kick at goal was made by the team in that particular sequence. If no try was scored and no kick at goal was made by the team in sequence *i*, the label *y*_*i*_ = −1 was assigned. Then, since the label now identified the scoring/not scoring outcome, the events that relate to the team scoring—Try scored (event ID = 11) and Kick at goal (event ID = 6)—were removed from the event sequences.

In the second, which we call the conceding dataset, we consider the case in which the sequences are from the team’s conceding perspective, or equivalently, from the opposition teams’ scoring perspective. In the conceding dataset, the label *y*_*i*_ = +1 was assigned to sequence *i* if a try was scored or a kick at goal was made by the *opposition* team in that particular sequence. If no try was scored and no kick at goal was made by the *opposition* team in sequence *i*, the label *y*_*i*_ = −1 was assigned. The list of events for the original delimited, scoring and conceding datasets are presented in Table 3. Then, since the label now identified the scoring/not scoring outcome, the events that relate to the *opposition* team scoring—Try scored (event ID = 11) and Kick at goal (event ID = 6)—were removed from the event sequences.

**Table 3:** Event lists for the original, scoring and conceding datasets.

event ID	original	scoring	conceding
1	Restart Received	Restart Received	Restart Received
2	Phase	Phase	Phase
3	Breakdown	Breakdown	Breakdown
4	Kick in Play	Kick in Play	Kick in Play
5	Penalty Conceded	Penalty Conceded	Penalty Conceded
6	Kick at Goal		Kick at Goal
7	Quick Tap	Quick Tap	Quick Tap
8	Line-out	Line-out	Line-out
9	Error	Error	Error
10	Scrum	Scrum	Scrum
11	Try Scored		Try Scored
12	Line Breaks	Line Breaks	Line Breaks
13	O-Restart Received	O-Restart Received	O-Restart Received
14	O-Phase	O-Phase	O-Phase
15	O-Breakdown	O-Breakdown	O-Breakdown
16	O-Kick in Play	O-Kick in Play	O-Kick in Play
17	O-Penalty Conceded	O-Penalty Conceded	O-Penalty Conceded
18	O-Kick at Goal	O-Kick at Goal	
19	O-Quick Tap	O-Quick Tap	O-Quick Tap
20	O-Line-out	O-Line-out	O-Line-out
21	O-Error	O-Error	O-Error
22	O-Scrum	O-Scrum	O-Scrum
23	O-Try Scored	O-Try Scored	
24	O-Line Breaks	O-Line Breaks	O-Line Breaks
label	-	Points Scored	O-Points Scored
	*n* = 490	*n*_+_ = 86, *n*_−_ = 404	*n*_+_ = 44, *n*_−_ = 446

**Fig 4:**
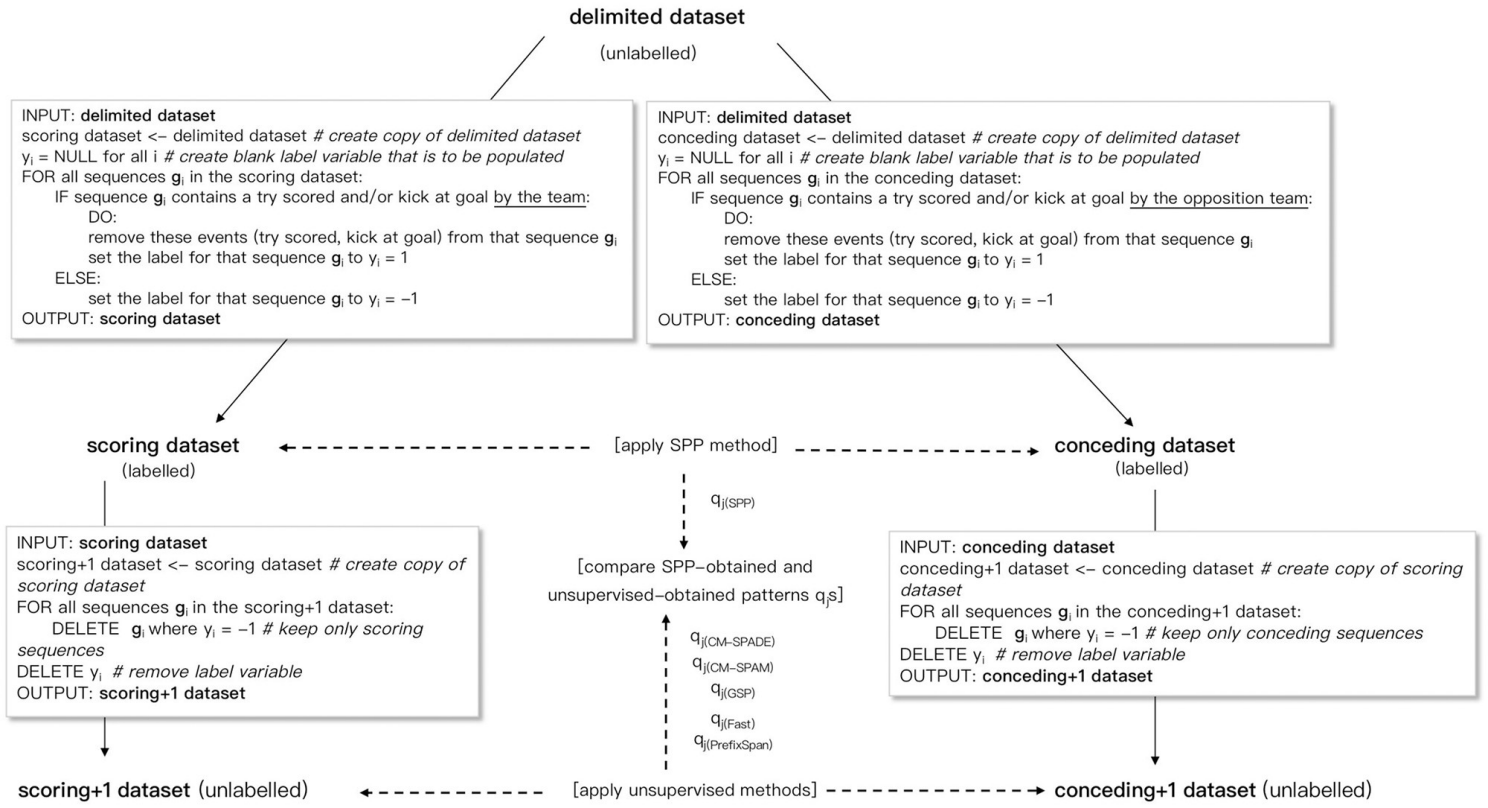
Illustration of dataset creation and experimental approach. Illustration of the procedures to create the datasets from the original delimited dataset to be used in the experiments and to compare the unsupervised and supervised SPM methods.

The process to create the scoring and conceding datasets from the original delimited dataset is shown in the upper half of Fig 4.

The SPP algorithm (software is available at https://github.com/takeuchi-lab/SafePatternPruning) was applied to the scoring and conceding datasets.

As a basis for comparison, we compared the patterns (*q*_*j*_s) obtained by SPP with those obtained by the following unsupervised methods: PrefixSpan, CM-SPAM, CM-SPADE, GSP and Fast. The SPMF pattern mining package [44] (v2.42c) was used to apply the five unsupervised SPM methods to our dataset. Since the unsupervised methods use unlabeled data, although support values of the patterns of play can be obtained (how many times a particular pattern occurred in the dataset), weights for the patterns cannot. For a more fair comparison between the unsupervised methods and SPP, we assume that the unsupervised methods have prior knowledge of the sequence labels. Thus, the unsupervised methods were applied to subsets of the scoring and conceding datasets, partitioned based on the label. The first subset, which we call the “scoring+1” dataset, contained only the sequences in which the team actually scored, and the second, the “conceding+1” dataset, contained only the sequences in which the team actually conceded (i.e., the opposition team scored).

The dataset creation process and comparative approach is presented in Fig 4.

#### Obtaining pattern weights with safe pattern pruning

As mentioned, our data consisted of sequences comprised of events from Table 2, labeled with an outcome: either +1 or −1, e.g.

−1 22 22 17

+1 8 11 2 6

−1 1 2 3 2 9

−1 20 21

−1 10 10 2 3 2 3 2 3 2 3 2 17

+1 8 11 2 6

−1 1 2 3 2 3 9

−1 22 16 2

−1 13 14 16

…

We used SPP to identify patterns that discriminate between outcome +1 and outcome −1. For instance, in the dataset above, it would appear that subsequence [2 3 2] is potentially a discriminative pattern, since it appears in three sequences that are labeled with −1 but does not appear in any sequences that are labeled with 1. Pattern [11 2 6] also appears to be a discriminative pattern since it appears in two sequences with label 1 and in none labeled with −1. In SPP, after performing safe screening and pruning, each remaining pattern in a sequence is multiplied by weights, e.g., *w*_1_ [2 3 2] + *w*_2_ [11 2 6]…, and then an optimization model solves for these weights.

SPP uses a classifier based on a sparse linear combinations of patterns, which can be written as

$$\begin{array}{c} f\left(g_{i};Q\right)=\sum_{q_{j}\in Q}w_{j}I\left(q_{j}⊑g_{i}\right)+b, \end{array}$$ (1)

where *I*(⋅) is an indicator function that takes the value 1 if sequence ***g***_*i*_ contains pattern ***g***_*i*_ and 0 other otherwise; and $w_{j}\in R$ and b∈R are parameters of the linear model that are estimated by solving the following minimization problem (as well as its dual maximization problem):

$$\begin{array}{c} \min_{w,b}\sum_{i\in [n]}\ell \left(y_{i},f\left(g_{i};Q\right)\right)+\lambda {∥w∥}_{1}, \end{array}$$ (2)

where ***w*** = [*w*_1_, …, *w*_*d*_]^⊤^ is a vector of weights, ℓ is a loss function and λ > 0 is a regularization parameter that can be tuned by cross-validation. Note that, due to the permutations in terms of the number of potential patterns of play, the size of Q is large in general. However, SPP’s pruning criterion reduces the size of Q by removing unnecessary patterns from the original pattern tree. The minimization problem (1) was, in the present study, solved with an L1-regularised L2-Support Vector Machine (the default option in the S3P classifier command line options https://github.com/takeuchi-lab/S3P-classifier), with 10-times 10-fold cross-validation used to tune the regularization parameter, lambda. The maximum pattern length parameter (option -L in the S3P classifier command line options) was set to 20. The feature vector ***x***_*i*_ = [*x*_*i*1_, *x*_*i*2_, …, *x*_*id*_] is defined for the *i*th sequence ***g***_*i*_ as

$$\begin{array}{c} x_{ij}=I\left(q_{j}⊑g_{i}\right),\;j=1,\ldots ,\left|Q\right|. \end{array}$$ (3)

In other words, the feature vectors, ***x***_*i*_ = [*I*(***q***_1_ ⊑ ***g***_*i*_), *I*(***q***_2_ ⊑ ***g***_*i*_), …, *I*(***q***_*d*_ ⊑ ***g***_*i*_)], are binary variables that take the respective values 1 or 0 based on whether or not pattern ***q***_*j*_ is contained within sequence ***g***_*i*_. In a two-class problem, the squared hinge-loss function *ℓ*(*y*, *f*(***x***_*i*_)) = max{0, 1 − *yf*(***x***_*i*_)}^2^ is used, and the optimization problem (2) becomes:

$$\begin{array}{c} \min_{w,b}\sum_{i\in [n]}\max{\left\{0,1-y_{i}\left(w^{⊤}x_{i}+b\right)\right\}}^{2}+\lambda {∥w∥}_{1}, \end{array}$$ (4)

Discriminative patterns are those that have positive weights (in absolute terms) in the optimal solution to (4), i.e., those patterns that have weights that are non-zero. As mentioned, SPP uses safe screening to remove weights that are guaranteed to be zero at the optimal solution, prior to solving the optimization problem (see S1 Appendix for more details.

In the results section below, in order to exclude patterns that may have occurred merely by chance, we have not reported obtained patterns (*q*_*j*_s) that had support values of less than five. In the case of the patterns obtained by the unsupervised methods, the top five patterns with the largest support values are reported. In the case of the SPP-obtained patterns, the top five patterns with the largest positive *w*_*j*_ values were recorded. In addition, we restricted our analysis to patterns of play that had the highest positive weights. For the scoring dataset, this means the patterns that had a positive contribution to the team scoring. For the conceding dataset, this means the patterns that had a positive contribution to opposition teams scoring. In other words, for the sake of brevity, we did not consider the patterns that had the highest contribution to “not scoring” and “not conceding.”

## Results and discussion

### Analysis of sequence lengths

**Fig 5:**
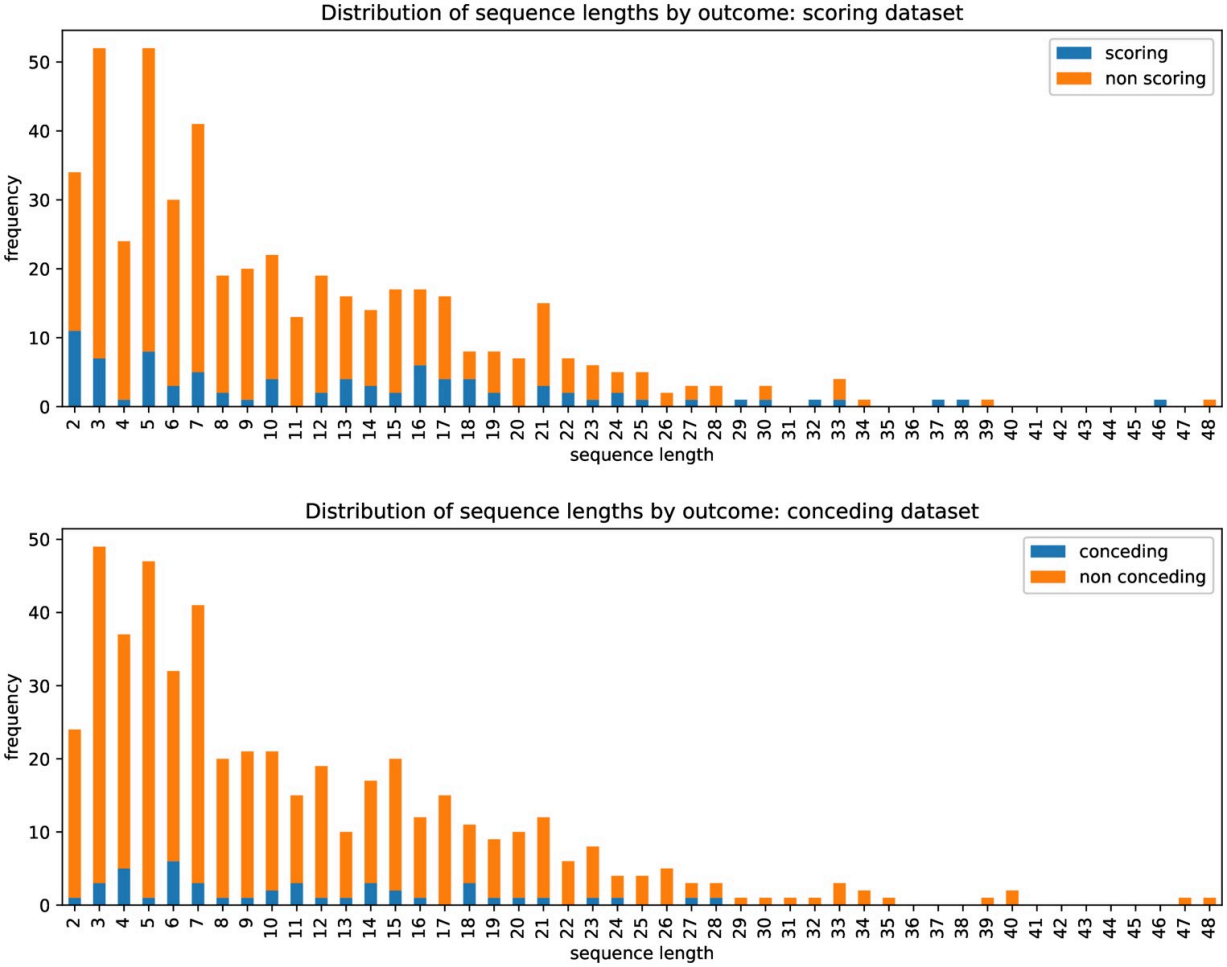
Sequence length distributions. Distribution of sequence lengths by points-scoring outcome for the scoring and conceding datasets. Sequence length is defined as the number of events in each sequence (excluding the outcome label).

The average sequence length in the scoring and conceding datasets was 10.6 and 10.8 events, respectively. The shortest sequence in both datasets contained two events, and the longest contained 48 events (Table 4). The slight difference in average sequence length between the scoring and conceding datasets is a result of the removal of the try scored and kick at goal events from the sequences in order to create the sequence outcome label (as was mentioned in the Materials and methods section above). The sequence length distributions (Fig 5) are positively skewed and, based on Shapiro-Wilk tests, non-normal. By comparing these distributions, it is clear that the number of sequences in which points were scored was higher in the scoring dataset than the conceding dataset, which reflects this particular team’s strength in the 2018 season. From the team’s scoring perspective, 86 out of the 490 passages of play (18%) resulted in points being scored by the team, while from the team’s conceding perspective, 44 out of the 490 passages of play (9%) resulted in points being conceded. The sequences in which the team scored were slightly longer, containing an average of 12.8 events compared to sequences in which the team didn’t score, which contained an average of 10.2 events. The sequences in which the team conceded and did not concede contained an average of 11.2 events and 10.8 events, respectively.

**Table 4:** Descriptive statistics for the scoring and conceding datasets.

	scoring	conceding
Mean	10.6	10.8
Standard deviation	7.8	7.9
Minimum	2	2
25th percentile	5	5
Median	8	8
75th percentile	15	15
Maximum	48	48
Skewness	1.3	1.4

### Identification of important patterns of play using SPP

SPP initially obtained 93 patterns when applied to the scoring dataset, of which 75 had support of five or higher. Of these 75 patterns of play, 38 had a positive weight (*w*_*j*_ > 0). The 75 patterns with minimum support of five contained an average of 4.5 events, and the 38 patterns with positive weights contained an average of 5.4 events. The longest obtained pattern in the scoring dataset contained 16 events.

Applying SPP to the conceding dataset resulted in a total of 72 patterns, of which 51 had support of five or higher. Of these 51 patterns of play, 31 had a positive weight (*w*_*j*_ > 0). The 51 patterns with minimum support of 5 contained an average of 3.8 events, and the 31 patterns with positive weights contained an average of 4.4 events. The longest obtained pattern in the conceding dataset contained 15 events.

The five patterns that discriminated the most between scoring and non-scoring outcomes (i.e., the patterns with the largest positive weight values) were obtained by applying SPP to the scoring dataset, and are listed along with their weight values and odds ratios in Table 5. In the results tables, the notation [*p*] × *n* indicates that pattern *p* is repeated *n* times. We also include the odds ratio (OR) for each pattern (simply the exponential of the weight), which aids in interpretation by providing a value that compares the cases where a sequence contains a particular pattern, and when it does not.

**Table 5:** Top five SPP-obtained patterns that discriminated the most between scoring and non-scoring outcomes.

pattern (*q*_*j*_)	pattern description	support	weight	OR
12	Line break	77	0.919	2.506
8 2	Line-out, Phase	71	0.808	2.242
2 3 4 2 3	Phase, Breakdown, Kick in play, Phase, Breakdown	9	0.796	2.217
2 3 2 3 2 3 2 3 4	[Phase, Breakdown] × 4, Kick in play	9	0.732	2.079
13 14 15 14 15 16 14 2 3	O-Restart received, [O-Phase, O-Breakdown] × 2, O-Kick in play, Phase, Breakdown	6	0.710	2.033

The pattern in the scoring dataset with the highest weight value (0.919), which discriminated the most between scoring and non-scoring sequences, was a pattern consisting of a single line break event (event id 12). The OR for the line break pattern is *e*^0.919^ = 2.506, meaning that the team is 2.5 times more likely to score when a line break is made in a sequence of play than if a line break is not made in a sequence of play. This makes sense since line breaks, which involve breaking through an opposition team’s line of defense (see the top-right image in Fig 2), generally advance the attacking team forward and are thus expected to create possible scoring opportunities. A line-out followed by phase play (8 2) was the second most discriminative pattern between scoring and not scoring, with a weight of 0.808 and an OR of 2.242, indicating that the team is 2.2 times more likely to score when a line-out followed by a phase occurs in a sequence of play than if it does not. The third most discriminative pattern, 2 3 4 2 3 (*w* = 0.796, *OR* = 2.217), can be interpreted as a kick in play being made by the team and being re-gathered by the team, thus resulting in retained possession. The OR indicates that the team is 2.2 times more likely to score when this pattern occurs in a sequence of play than if it does not. The fourth most discriminative pattern, 2 3 2 3 2 3 2 3 4 (*w* = 0.732, *OR* = 2.079), represents four repeated phase-breakdown plays by the team, followed by the team making a kick in play. This pattern thus involves repeated retaining of possession before the team presumably gaining territory in the form of a kick. The OR indicates that the team is 2.1 times more likely to score when this pattern occurs in a sequence of play than if it does not. The fifth most discriminative pattern, 13 14 15 14 15 16 14 2 3 (*w* = 0.710, *OR* = 2.033), can be interpreted as the opposition team receiving a kick restart made by the team, attempting to exit their own territory via a kick but not finding touch, thus giving the ball back to the team from which they can potentially build phases and launch an attack. The OR indicates that the team is twice as likely to score when this pattern occurs in a sequence of play than if it does not.

The five patterns that discriminated the most between conceding and non-conceding outcomes were obtained by applying SPP to the conceding dataset, and are listed along with their weights and ORs in Table 6. A line break (event ID 24) (*w* = 0.613, *OR* = 1.846) being made by the opposition team was the pattern that discriminated most between sequences in which the team conceded (i.e., the opposition team scored) and sequences in which the team did not concede (i.e., the opposition team did not score). In other words, a line break by the opposition team was the pattern that discriminated the most between the group of sequences in which the opposition team scored and the group of sequences in which the opposition team did not score. The OR of 1.8 indicates that the opposition team is 1.8 times more likely to score when they make a line break in a sequence of play than if they do not. The weight (and OR) for the line break pattern was not as large as in the scoring dataset (*w* = 0.919 vs. *w* = 0.613), which suggests that the team had strong defense in this particular season. The second most discriminative pattern between conceding and non-conceding outcomes, 14 9 15 (*w* = 0.392, *OR* = 1.479), can be interpreted as the opposition team being in possession of the ball, the team making some form of error, and the opposition team regaining possession. The opposition team is 1.5 times more likely to score when this pattern occurs in a sequence of play than if it does not. The third most discriminative pattern between conceding and non-conceding outcomes was an opposition team line-out (*w* = 0.357, *OR* = 1.428). The opposition team is 1.4 times more likely to score if they have a line-out in a sequence of play than if they do not. The fourth (*w* = 0.339, *OR* = 1.403) and fifth (*w* = 0.261, *OR* = 1.299) most discriminative patterns for the conceding dataset represent repeated phase and breakdown play, which results in retained possession and the building of pressure. The fifth pattern, for example, is one in which the opposition team makes over six repeated consecutive phases and breakdowns.

**Table 6:** Top five SPP-obtained patterns that discriminated the most between conceding and non-conceding outcomes.

event id pattern (*q*_*j*_)	pattern description	support	weight	OR
24	O-Line break	32	0.613	1.846
14 9 15	O-Phase, Error, O-Breakdown	10	0.392	1.479
20	O-Line-out	86	0.357	1.428
15 15 14 15	O-Breakdown, O-Breakdown, O-Phase, O-Breakdown	5	0.339	1.403
15 14 15 14 15 14 15 14 15 14 15 14 15	[O-Breakdown, O-Phase] × 6, O-Breakdown	16	0.261	1.299

### Comparing the SPP-obtained patterns with those obtained by the unsupervised methods

The five patterns with the highest support for the scoring+1 and conceding+1 datasets, obtained by applying each of the five unsupervised methods (PrefixSpan, CM-SPAM, CM-SPADE, GSP and Fast), are shown in Tables 7 and 8, respectively.

**Table 7:** Top five PrefixSpan-obtained patterns with the largest support: Scoring+1 dataset.

PrefixSpan	CM-SPAM	CM-SPADE	GSP	Fast	support
2	2	2	2	2	84
2 3	3	3	3	3	60
3	2 3	2 3	2 3	2 3	60
2 2	2 2	3 2	2 2	2 2	59
2 3 2	2 3 2	2 2	3 2	3 2	59

**Table 8:** Top five PrefixSpan-obtained patterns with the largest support: Conceding+1 dataset.

PrefixSpan	CM-SPAM	CM-SPADE	GSP	Fast	support
14	14	14	14	14	39
14 15	15	15	15	15	33
15	14 15	14 15	14 15	14 15	33
14 14	14 14	15 14	14 14	14 14	29
14 15 14	14 15 14	14 14	15 14	15 14	29

Tables 7 and 8 show that only common events and patterns were detected by the unsupervised methods, i.e., patterns containing breakdowns, phases, or both. Repeated breakdown and phase play means that a team can generally retain possession of the ball and build pressure pressure (see the middle and bottom images on the right-hand side of Fig 2). While some of the patterns identified by SPP also contained repeated breakdown and phase play, they were generally longer and also contained other events. The patterns obtained by the unsupervised methods are not particularly useful for coaches or performance analysts since they merely reflect common, repeated patterns rather than interesting patterns. By using passage of play event sequences that are labelled with scoring or conceding outcomes, through the computed weights, SPP is also able to provide a measure of the degree to which particular patterns discriminate between these outcomes. In addition, compared to the unsupervised methods, the supervised SPP method obtained a greater variety of patterns of play (i.e., not only those containing breakdowns and/or phases) and also discovered more sophisticated patterns that can be readily interpreted by coaches or performance analysts.

### Discussion

By considering both the scoring and conceding perspectives of the team, insight was able to be obtained that would be useful to both the team as well as opposition teams that are due to play the team. For both the team and their opposition teams during the 2018 season, line breaks were found to be most associated with scoring. For both the team and their opposition teams, line-outs were found to be more beneficial in generating scoring opportunities than scrums. This result is consistent with [41], who found that line-outs followed by a driving maul are common approaches to scoring tries (albeit in a different competition, Super Rugby), and with [45], who found that around one-third of tries came from line-outs in the Japan Top League in 2003 to 2005—the highest of any try source. As well as creating line-outs or perhaps prioritising them over scrums, for opposition teams playing the team, effective strategies may include aiming to maintain possession through repeated phase-breakdown play (over six repetitions), shutting down the team’s ability to regain kicks, and ensuring that touch is found on exit plays from kick restarts made by the team.

As mentioned, compared to the unsupervised methods, the supervised SPP method obtained a greater variety of patterns that consisted of a greater variety of events. The unsupervised methods only generated patterns consisting of either phases, breakdowns, or both, which are very frequent and repetitive patterns but are not of use to coaches or performance analysts. As well as containing a greater variety of events, the patterns obtained by SPP were more complex in terms of the patterns of play that were identified. For instance, through one of the SPP-obtained patterns, an opposition team could identify that they could be punished by the team for a failed exit play. The pattern involving the team making and regaining a kick in play (which was shown to discriminate between scoring and non-scoring outcomes for the team) is another example of a complex pattern of play that was identified by SPP. The superiority of SPP over the unsupervised methods is likely due to the discriminative nature of SPP as well as the safe screening and pattern pruning mechanisms of SPP, which prune out irrelevant sequential patterns and model weights in advance.

## Conclusions and future work

In this study, a supervised sequential pattern mining (SPM) method called safe pattern pruning (SPP) was applied to data from professional rugby union in Japan that consisted of sequences in the form of passages of play that were labelled with points scoring outcomes. The obtained results suggest that the SPP model was useful in detecting complex patterns (patterns of play) that are important to scoring outcomes. SPP was able to identify relatively sophisticated, discriminative patterns of play, which made sense when interpreted, and which are potentially useful for coaches and performance analysts for own- and opposition-team analysis in order to identify vulnerabilities and tactical opportunities. The approach highlighted the potential utility of supervised SPM as an analytical framework for performance analysis in sport, and more specifically, the potential usefulness of SPM methods for performance analysis in rugby.

Although the results obtained are encouraging, a limited amount of data from one sport was used. Also, spatial information such as field position was not available in the data, and this may have improved the analysis. Although the team that performed a particular event was used in our analysis, which player performed particular events was not considered—this may be interesting to investigate in future work. One limitation of SPP is that, although it considers the order of events within the sequences, the method does not consider the order of sequences within matches, which could also be informative (e.g., a particular pattern occurring in the second half of a match may be more important than if it occurs in the first half). Furthermore, although SPP was useful for the specific dataset in this study, its usefulness is to some degree dependent on the structure of the input data and the specific definition of the sequences and labels. For instance, as mentioned earlier, applying the approach to a dataset that consists of entire matches as sequences and match win/loss outcomes does not tend to produce interesting results since it is self-evident that sequences that contain more scoring events will be more associated with wins, and so SPP would simply pick up the scoring events in such datasets. In future work, it would be interesting to apply the approach to a larger amount of data from rugby, as well as to similarly structured datasets in other sports in order to confirm its efficacy.

## Supporting information

S1 Dataset The delimited sequence data that is described in this paper is available 452 on GitHub: https://github.com/rorybunker/rugby-sequences. (TXT)

S1 Appendix Safe screening and regularization path initialization. (PDF)

## References

[1] Hughes MD, Bartlett RM. The use of performance indicators in performance analysis. Journal of sports sciences. 2002 Jan 1;20(10):739–54. doi: 10.1080/026404102320675602 12363292

[2] Agrawal R, Srikant R. Mining sequential patterns. In: Proceedings of the eleventh international conference on data engineering. 1995 Mar 6 (pp. 3–14). IEEE.

[3] Mabroukeh NR, Ezeife CI. A taxonomy of sequential pattern mining algorithms. ACM Computing Surveys (CSUR). 2010 Dec 3;43(1): 1–41. doi: 10.1145/1824795.1824798

[4] Wang K, Xu Y, Yu JX. Scalable sequential pattern mining for biological sequences Proceedings of the thirteenth ACM international conference on Information and knowledge management. 2004 Nov 13 (pp. 178–187).

[5] Ho J, Lukov L, Chawla S. Sequential pattern mining with constraints on large protein databases. In Proceedings of the 12th international conference on management of data (COMAD). 2005 (pp. 89–100).

[6] Exarchos TP, Papaloukas C, Lampros C, Fotiadis DI. Mining sequential patterns for protein fold recognition Journal of Biomedical Informatics. 2008 Feb 1;41(1):165–79 doi: 10.1016/j.jbi.2007.05.004 17573243

[7] Hsu CM, Chen CY, Liu BJ, Huang CC, Laio MH, Lin CC, et al. Identification of hot regions in protein-protein interactions by sequential pattern mining BMC bioinformatics. 2007 May;8(5):1–5. doi: 10.1186/1471-2105-8-S5-S8 17570867 PMC1892096

[8] Garboni C, Masseglia F, Trousse B. Sequential pattern mining for structure-based XML document classification. In International Workshop of the Initiative for the Evaluation of XML Retrieval. 2005 Nov 28 (pp. 458–468).

[9] Feng J, Xie F, Hu X, Li P, Cao J, Wu X. Keyword extraction based on sequential pattern mining. In proceedings of the third international conference on internet multimedia computing and service. 2011 Aug 5 (pp. 34–38).

[10] Xie F, Wu X, Zhu X. Document-specific keyphrase extraction using sequential patterns with wildcards. In: 2014 IEEE International Conference on Data Mining 2014 Dec 14 (pp. 1055–1060). IEEE.

[11] Xie F, Wu X, Zhu X. Efficient sequential pattern mining with wildcards for keyphrase extraction. Knowledge-Based Systems. 2017 Jan 1;115:27–39. doi: 10.1016/j.knosys.2016.10.011

[12] Yap GE, Li XL, Philip SY. Effective next-items recommendation via personalized sequential pattern mining. In: International conference on database systems for advanced applications 2012 Apr 15 (pp. 48–64). Springer, Berlin, Heidelberg.

[13] Salehi M, Kamalabadi IN, Ghoushchi MB. Personalized recommendation of learning material using sequential pattern mining and attribute based collaborative filtering. Education and Information Technologies. 2014 Dec;19(4):713–35. doi: 10.1007/s10639-012-9245-5

[14] Ceci M, Lanotte PF, Fumarola F, Cavallo DP, Malerba D. Completion time and next activity prediction of processes using sequential pattern mining. In International Conference on Discovery Science 2014 Oct 8 (pp. 49–61). Springer, Cham.

[15] Wright AP, Wright AT, McCoy AB, Sittig DF. The use of sequential pattern mining to predict next prescribed medications. Journal of biomedical informatics. 2015 Feb 1;53:73–80. doi: 10.1016/j.jbi.2014.09.003 25236952

[16] Tsai CY, Lai BH. A location-item-time sequential pattern mining algorithm for route recommendation. Knowledge-Based Systems. 2015 Jan 1;73:97–110. doi: 10.1016/j.knosys.2014.09.012

[17] Tarus JK, Niu Z, Kalui D. A hybrid recommender system for e-learning based on context awareness and sequential pattern mining. Soft Computing. 2018 Apr;22(8):2449–61. doi: 10.1007/s00500-017-2720-6

[18] Fournier-Viger P, Lin JCW, Kiran RU, Koh YS, Thomas R. A survey of sequential pattern mining. Data Science and Pattern Recognition. 2017;1(1):54–77.

[19] Yao H, Hamilton HJ, Butz, CJ. A foundational approach to mining itemset utilities from databases. In: Proceedings of the 2004 SIAM International Conference on Data Mining 2004 Apr 22 (pp. 482–486). Society for Industrial and Applied Mathematics.

[20] Gan W, Lin JC, Zhang J, Fournier-Viger P, Chao H, Yu P. Fast utility mining on sequence data. IEEE transactions on cybernetics. 2020 Feb 28;51(2):487–500. doi: 10.1109/TCYB.2020.2970176 32142464

[21] Yin J, Zheng Z, Cao L. USpan: an efficient algorithm for mining high utility sequential patterns. Proceedings of the 18th ACM SIGKDD International Conference on Knowledge Discovery and Data Mining 2012 Aug 12 (pp. 660–668).

[22] Wang J, Huang J, Chen Y. On efficiently mining high utility sequential patterns. Knowledge and Information Systems. 2016 Nov;49(2):597–627. doi: 10.1007/s10115-015-0914-8

[23] Lin JC, Srivastava G, Li Y, Hong T, Wang S. Mining High-Utility Sequential Patterns in Uncertain Databases. In: 2020 IEEE International Conference on Big Data (Big Data) 2020 Dec 10 (pp. 5373–5380). IEEE.

[24] Srivastava G, Lin JC, Zhang X, Li Y. Large-scale high-utility sequential pattern analytics in Internet of things. IEEE Internet of Things Journal. 2020 Sep 25.

[25] Wu JM, Srivastava G, Wei M, Yun U, Lin JC. Fuzzy high-utility pattern mining in parallel and distributed Hadoop framework. Information Sciences. 2021 Apr 1;553:31–48. doi: 10.1016/j.ins.2020.12.004

[26] Srikant R, Agrawal R. Mining sequential patterns: Generalizations and performance improvements. In: International Conference on Extending Database Technology 1996 Mar 25 (pp. 1–17). Springer, Berlin, Heidelberg

[27] Agarwal R, Srikant R. Fast algorithms for mining association rules. In: Proc. 20th int. conf. very large data bases, VLDB 1994 Sep 12 (Vol. 1215, pp. 487–499).

[28] Zaki MJ. SPADE: An efficient algorithm for mining frequent sequences Machine learning. 2001 Jan;42(1):31–60.

[29] Ayres J, Flannick J, Gehrke J, Yiu T. Sequential pattern mining using a bitmap representation. In: Proceedings of the Eighth ACM SIGKDD International Conference on Knowledge Discovery and Data Mining 2002 Jul 23 (pp. 429–435).

[30] Pei J, Han J, Mortazavi-Asl B, Wang J, Pinto H, Chen Q, et al. Mining sequential patterns by pattern-growth: The prefixspan approach. IEEE Transactions on knowledge and data engineering. 2004 Oct 4;16(11):1424–40. doi: 10.1109/TKDE.2004.77

[31] Fournier-Viger P, Gomariz A, Campos M, Thomas R. Fast vertical mining of sequential patterns using co-occurrence information. In: Pacific-Asia Conference on Knowledge Discovery and Data Mining 2014 May 13 (pp. 40–52). Springer, Cham.

[32] Salvemini E, Fumarola F, Malerba D, Han J. Fast sequence mining based on sparse id-lists. In International Symposium on Methodologies for Intelligent Systems 2011 Jun 28 (pp. 316–325). Springer, Berlin, Heidelberg.

[33] Nakagawa K, Suzumura S, Karasuyama M, Tsuda K, Takeuchi I. Safe pattern pruning: An efficient approach for predictive pattern mining. In: Proceedings of the 22nd ACM SIGKDD international conference on knowledge discovery and data mining 2016 Aug 13 (pp. 1785–1794).

[34] Sakuma T, Nishi K, Kishimoto K, Nakagawa K, Karasuyama M, Umezu Y, et al. Efficient learning algorithm for sparse subsequence pattern-based classification and applications to comparative animal trajectory data analysis. Advanced Robotics. 2019 Feb 16;33(3-4):134–52. doi: 10.1080/01691864.2019.1571438

[35] Ghaoui LE, Viallon V, Rabbani T. Safe feature elimination for the lasso and sparse supervised learning problems. arXiv preprint arXiv:1009.4219. 2010 Sep 21.

[36] La Puma I, de Castro Giorno FA. Ontology-Based Data Mining Approach for Judo Technical Tactical Analysis. In: The third international conference on computing technology and information management (ICCTIM2017) 2017 Dec 8 (p. 90).

[37] Hrovat G, Fister I Jr, Yermak K, Stiglic G, Fister I. Interestingness measure for mining sequential patterns in sports. Journal of Intelligent & Fuzzy Systems. 2015 Jan 1;29(5):1981–94. doi: 10.3233/IFS-151676

[38] Decroos T, Van Haaren J, Davis J. Automatic discovery of tactics in spatio-temporal soccer match data. In: Proceedings of the 24th ACM SIGKDD International Conference on Knowledge Discovery & Data Mining 2018 Jul 19 (pp. 223–232).

[39] Carter A, Potter G. The 1995 rugby world cup finals. 187 tries. In Hughes, M. (Ed.) (2001). Notational Analysis of Sport III. University of Wales Institute Cardiff. Cardiff, Wales UWIC 2001 (pp. 224–229).

[40] van Rooyen KM, Noakes DT. Movement time as a predictor of success in the 2003 Rugby World Cup Tournament. International Journal of Performance Analysis in Sport. 2006 Jun 1;6(1):30–9. doi: 10.1080/24748668.2006.11868353

[41] Coughlan M, Mountifield C, Sharpe S, Mara JK. How they scored the tries: applying cluster analysis to identify playing patterns that lead to tries in super rugby. International Journal of Performance Analysis in Sport. 2019 May 4;19(3):435–51. doi: 10.1080/24748668.2019.1617018

[42] Watson N, Hendricks S, Stewart T, Durbach I. Integrating machine learning and decision support in tactical decision-making in rugby union. Journal of the Operational Research Society. 2020 Jul 31:1–2. doi: 10.1080/01605682.2020.1779624

[43] Liu T, Fournier-Viger P, Hohmann A. Using diagnostic analysis to discover offensive patterns in a football game. In: Recent Developments in Data Science and Business Analytics 2018 (pp. 381–386). Springer, Cham.

[44] Fournier-Viger P, Gomariz A, Gueniche T, Soltani A, Wu CW, Tseng VS. Spmf: a java open-source pattern mining library. J. Mach. Learn. Res. 2014 Jan;15(1):3389–93.

[45] Sasaki K, Furukawa T, Murakami J, Shimozono H, Nagamatsu M, Miyao M, et al. Scoring profiles and defense performance analysis in Rugby Union. International Journal of Performance Analysis in Sport. 2007 Oct 1;7(3):46–53. doi: 10.1080/24748668.2007.11868409

