# Supplementary material for: Supervised sequential pattern mining of event sequences in sport to identify important patterns of play: An application to rugby union
Source: PLoS One. 2021 Sep 23;16(9):e0256329. doi: 10.1371/journal.pone.0256329 (PMC8460049; doi:10.1371/journal.pone.0256329)
Supplement: S1 Appendix — (PDF) [file pone.0256329.s002.pdf]

S1 Appendix. Safe Screening and Regularization Path Initialization. Some weights are removed prior to solving (4) using safe screening, which corresponds to finding j such that $w_j = 0$ in the optimal solution $\boldsymbol{w}^* := [w_1^*, \ldots, w_d^*]^\top$ in the optimization problem (4). Such w_j do not affect the optimal solution even if they are removed prior to solving the optimization problem. In the optimal solution, the $\boldsymbol{w}^*$ of the optimization problem (2), a set of j such that $|w_j^*| > 0$ is called the active set, and is denoted as $\mathcal{A} \subseteq \mathcal{Q}$. In this case, even if only the patterns included in $\mathcal{A}$ are used, the same optimal solution—as when using all patterns—can be obtained. Thus, if one solves

$$(\boldsymbol{w}'^*_{\mathcal{A}}, b'^*) := \operatorname*{argmin}_{\boldsymbol{w}, b} \sum_{i \in [n]} \ell(y_i, f(\boldsymbol{g}_i; \{\boldsymbol{q}\}_{i \in \mathcal{A}})) + \lambda \|\boldsymbol{w}\|_1, \tag{1}$$

then it is guaranteed that $\boldsymbol{w}^* = \boldsymbol{w}'^*_{\mathcal{A}}$ and $b^* = b'^*$.

In practice, the λ parameter is found based on a model selection technique such as cross-validation. In model selection, a sequence of solutions, a so-called regularization path, with various penalty parameters must be trained. The regularization path of the problem (2), $\{\lambda_0, \lambda_1, \ldots, \lambda_K\}$, is usually computed with decreasing λ, because sparser solutions are obtained for larger λ.

To compute the regularization path, the initial values are set to $\boldsymbol{w}^* \longleftarrow \mathbf{0}$, $b^* \longleftarrow \bar{y}$ (where $\bar{y}$ is the sample mean of $\{y_i\}_{i \in [n]}$) and $\lambda_0 \longleftarrow \lambda_{\max}$ (see [1] for how $\lambda_{\max}$ is calculated, and for further details of the SPP method as well as its safe-screening mechanism and pruning criterion).

Reference

1. Sakuma T, Nishi K, Kishimoto K, Nakagawa K, Karasuyama M, Umezu Y, Kajioka S, Yamazaki SJ, Kimura KD, Matsumoto S, Yoda K. Efficient learning algorithm for sparse subsequence pattern-based classification and applications to comparative animal trajectory data analysis. Advanced Robotics. 2019 Feb 16;33(3-4):134-52.
